# Neural oscillations underlying selective attention follow sexually divergent developmental trajectories during adolescence

**DOI:** 10.1016/j.dcn.2021.100961

**Published:** 2021-05-07

**Authors:** Brittany K. Taylor, Jacob A. Eastman, Michaela R. Frenzel, Christine M. Embury, Yu-Ping Wang, Vince D. Calhoun, Julia M. Stephen, Tony W. Wilson

**Affiliations:** a Institute for Human Neuroscience, Boys Town National Research Hospital, Boys Town, NE, USA; b Department of Psychology, University of Nebraska Omaha, Omaha, NE, USA; c Department of Biomedical Engineering, Tulane University, New Orleans, LA, USA; d Mind Research Network, Albuquerque, NM, USA; e Tri-institutional Center for Translational Research in Neuroimaging and Data Science (TReNDS), Georgia State University, Georgia Institute of Technology, Emory University, Atlanta, GA, USA

**Keywords:** Development, Flanker effect, Magnetoencephalography (MEG), Oscillations, Sex effects

## Abstract

Selective attention processes are critical to everyday functioning and are known to develop through at least young adulthood. Although numerous investigations have studied the maturation of attention systems in the brain, these studies have largely focused on the spatial configuration of these systems; there is a paucity of research on the neural oscillatory dynamics serving selective attention, particularly among youth. Herein, we examined the developmental trajectory of neural oscillatory activity serving selective attention in 53 typically developing youth age 9-to-16 years-old. Participants completed the classic arrow-based flanker task during magnetoencephalography, and the resulting data were imaged in the time-frequency domain. Flanker interference significantly modulated theta and alpha/beta oscillations within prefrontal, mid-cingulate, cuneus, and occipital regions. Interference-related neural activity also increased with age in the temporoparietal junction and the rostral anterior cingulate. Sex-specific effects indicated that females had greater theta interference activity in the anterior insula, whereas males showed differential effects in theta and alpha/beta oscillations across frontoparietal regions. Finally, males showed age-related changes in alpha/beta interference in the cuneus and middle frontal gyrus, which predicted improved behavioral performance. Taken together, these data suggest sexually-divergent developmental trajectories underlying selective attention in youth.

## Highlights

• A cohort of 9- to 16-year-olds completed a classic flanker task during MEG.
• There were developmentally-sensitive interference effects in key attention regions.
• Youth showed sexually-divergent patterns of age-related interference activity.
• Maturational differences among males supported improved task behavior.

## 1 Introduction

Attentional processes are critical to everyday functioning, as they allow cognitive resources to be directed to specific environmental or internal features. Selective attention is the ability to focus on specific stimuli while ignoring other competing or distracting information in the environment (Johnston and Dark, 1986; Kahneman et al., 2017; Posner and Petersen, 1990). Neurologically, selective attention is thought to involve the allocation of neural resources to specific target stimuli, while simultaneously inhibiting or suppressing resources dedicated to unattended stimuli (Dayan et al., 2000; Moore and Zirnsak, 2017; Parks and Madden, 2013; Petersen and Posner, 2012; Salo et al., 2017). Such neurocognitive abilities are known to develop throughout childhood and adolescence, with attentional function generally improving and becoming less variable with increasing age (Amso and Scerif, 2015; Petersen and Posner, 2012; Rothbart and Posner, 2015).

A number of neuropsychological assessments and behavioral tasks have been developed for measuring selective attention abilities, and several of these have been adapted for use in neuroimaging studies (e.g., Downing et al., 2001; Fan et al., 2007; Hopf et al., 2010; Keller et al., 2017; Popov et al., 2018). One such paradigm, the flanker task (Eriksen and Eriksen, 1974), requires individuals to selectively attend to a centrally located target image (often a left or right pointing arrow) that is flanked by an array of either congruently- or incongruently-oriented distractor stimuli. The conflict that arises from the presence of incongruent distractors is termed the “flanker effect,” and frequently leads to slowed reaction times as well as changes in neural activity during stimulus processing and decision making (Albrecht et al., 2009; Botvinick et al., 2004; Grent-’t-Jong et al., 2013; Heinrichs-Graham et al., 2018a). The behavioral flanker effect is less robust in young children (e.g., 4–6 years) and sometimes non-existent (McDermott et al., 2007), but it appears to gradually emerge during late childhood and then is sustained during adolescence and throughout adulthood (Gavin et al., 2019; McDermott et al., 2017; Segalowitz and Davies, 2004; Wiesman et al., 2020).

Given the trajectory of neural maturation within attention networks (Pozuelos et al., 2014; Rueda et al., 2004), the developmental sensitivity of the flanker effect is unsurprising. Briefly, functional MRI (fMRI) studies have repeatedly identified frontoparietal networks as central to adequate performance in the presence of attentional distractors, with greater frontal activity during conflict generally associated with decreased behavioral flanker effects (for a review, see Parks and Madden, 2013). In addition to prefrontal and parietal regions, the dorsal anterior cingulate cortex (dACC) and anterior insula are frequently highlighted in studies examining attentional distractors, and are believed to play a role in signaling conflict and possibly compensatory processing (Botvinick et al., 2004; Chaddock et al., 2012; Huyser et al., 2011; Parks and Madden, 2013; Vaidya et al., 2005). All of these regions are well-known to undergo dramatic structural changes through childhood and adolescence, with remarkable alterations in gray matter volume and thickness, white matter integrity, and interregional connectivity (Bunge et al., 2002; Casey et al., 2005; Dumontheil, 2016; Durston et al., 2006). Interestingly, there is also evidence that these brain regions follow sexually divergent maturational trajectories during puberty, as development within these regions has been shown to peak at different times in males versus females, which is likely linked to differences in the influence of pubertal hormones (Blakemore et al., 2010; Blakemore and Choudhury, 2006).

Despite mounting evidence of structural and functional developmental alterations across selective attention circuits during adolescence, the neural oscillatory dynamics within these circuits have been seldom studied in youth. Understanding the impact of development on these oscillatory dynamics is critical, as oscillatory activity at the population-level is known to be central to neural coding and information processing more generally (de Pasquale et al., 2010; Fries, 2005; Hipp et al., 2011; Wilson et al., 2016). Previous selective attention studies in adults have repeatedly shown increases in theta (4−8 Hz) and decreases in alpha (8−12 Hz) across a distributed network of frontoparietal regions (Lew et al., 2018, 2020; McDermott et al., 2017; Wiesman et al., 2020). Specifically, studies using magnetoencephalography (MEG) have shown that the increase in theta activity originates in dorsal and ventral frontal areas, while the alpha oscillations emerge from more posterior occipital and parietal regions (Lew et al., 2018; McDermott et al., 2017). These findings have been supported and extended by studies using flanker-like tasks in other contexts, including brain stimulation (McDermott et al., 2019; Spooner et al., 2019), clinical conditions known to affect attention function (Embury et al., 2018; Lew et al., 2018), and even aging (Wiesman et al., 2020) where alterations in these oscillatory dynamics were shown to covary with behavioral performance. Thus, despite extensive studies in adults and abundant evidence that the oscillatory dynamics serving other cognitive and motor processes are developmentally sensitive in youth (Embury et al., 2019; Heinrichs-Graham et al., 2018b; Taylor et al., 2020; Trevarrow et al., 2019; Wilson et al., 2010), there remains a paucity of work examining the developmental trajectories of the neural oscillatory dynamics serving selective attention in childhood and adolescence.

The goal of the present study was to identify the developmental trajectory of the neural oscillatory dynamics serving selective attention during late childhood and adolescence. We first mapped the developmental trajectories for the full sample, and then separately for males versus females to better gauge any sexually-divergent maturational trajectories. In accordance with prior literature (e.g., McDermott et al., 2017), we hypothesized that selective attention would be served by theta activity in frontoparietal regions, and by alpha activity in more posterior cortices. We expected that activity within frontoparietal regions would strengthen as a function of age, and that there would be sex-specific developmental effects based on prior literature showing sexually-divergent trajectories in other higher-order abilities like working memory (Embury et al., 2019) and abstract reasoning (Taylor et al., 2020).

## 2 Materials and methods

### 2.1 Participants

A total of 71 youth between the ages of 9- and 16-years-old (*M* = 13.15 years, *SD* = 1.94; 40 males; 5 left-handed) completed a Flanker task as part of the Developmental Chronnecto-Genomics (Dev-CoG) study (http://devcog.mrn.org; Stephen et al., 2021). All participants were recruited from the University of Nebraska Medical Center (UNMC) site. Exclusionary criteria included an inability to perform the task, any medical illness affecting CNS function, neurological or psychiatric disorder, history of head trauma, current substance abuse, any medication known to affect CNS function, and the MEG Laboratory’s standard exclusion criteria (e.g., dental braces, metal implants, battery operated implants, and/or any type of ferromagnetic implanted material). Parents of youth participants signed informed consent forms, and youth participants signed assent forms before proceeding with the study. All procedures were approved by the UNMC Institutional Review Board, and were in accordance with the Declaration of Helsinki.

### 2.2 MEG experimental paradigm

**Fig. 1:**
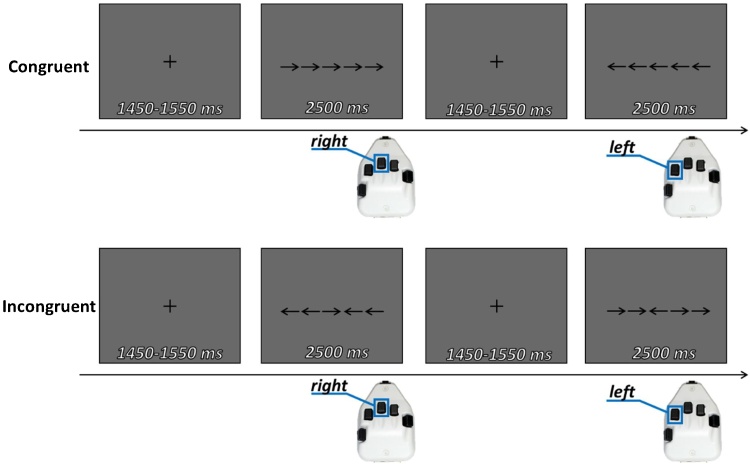
Structure of the flanker task utilized in this study. Each participant completed 100 congruent and 100 incongruent trials, which were balanced on the direction of the target arrow and pseudo-randomly presented. Button press responses are indicated below the trial.

Participants completed an arrow-based Flanker task previously used by our lab (e.g., (Embury et al., 2018; McDermott et al., 2017; Wiesman et al., 2020). Briefly, each trial began with a fixation cross presented in the center of the screen for a jittered duration of 1450−1550 ms (see Fig. 1). Then, an array of five centrally-presented arrows was presented for 2500 ms.

The center arrow could either be congruent (i.e., pointing the same direction as the flanking arrows), or incongruent (i.e., pointing the opposite direction of the flanking arrows). There were 200 total trials, equally split between congruent and incongruent conditions, pseudorandomly presented. Participants were given a button pad and instructed to press a button with their right index finger if the center arrow pointed left, or with their right middle finger if the center arrow pointed right. Participants were given a 30-second break at the halfway point of the task; the task lasted approximately 14 min. Standard data trimming procedures were used before examining accuracy or reaction time on the task. Namely, we examined each participant’s individual data and excluded trials in which response times exceeded 2.5 standard deviations from that individual’s mean response time (McDermott et al., 2017). The trimming procedure eliminated, on average, 2.86 % (*SD* = 1.03) of congruent trials, and 2.96 % (SD = 1.11) of incongruent trials from further analyses. The number of eliminated trials did not differ between conditions (*t*(71) = .60, *p* = .73).

### 2.3 MEG data acquisition

MEG recordings were conducted in a one-layer magnetically shielded room with active shielding engaged. Neuromagnetic responses were acquired with an Elekta/MEGIN MEG system with 306 magnetic sensors (204 planar gradiometers, 102 magnetometers; Elekta, Helsinki, Finland) using a bandwidth of 0.1–330 Hz, sampled continuously at 1 kHz. Each participant's data were individually corrected for head motion, and noise reduction was applied using the signal space separation method with a temporal extension (tSSS; Taulu and Simola, 2006; Taulu et al., 2005).

### 2.4 MEG coregistration and structural MRI processing

In preparation for the MEG measurement, four coils were attached to the participant's head and localized, together with the three fiducial points and scalp surface, using a 3-D digitizer (Fastrak 3SF0002, Polhemus Navigator Sciences, Colchester, VT, USA). Once the participant was positioned for MEG recording, an electric current with a unique frequency label (e.g., 322 Hz) was fed to each of the coils. This induced a measurable magnetic field and allowed each coil to be localized in reference to the sensors throughout the recording session. Since coil locations were also known in head coordinates, all MEG measurements could be transformed into a common coordinate system. With this coordinate system, each participant's MEG data were coregistered with their individual structural T1-weighted MRI data prior to source space analyses using BESA MRI (Version 2.0; BESA GmbH, Gräfelfing, Germany). Structural T1-weighted MR images were acquired using a Siemens Skyra 3 T MRI scanner with a 32-channel head coil and a MP-RAGE sequence with the following parameters: TR =2400 ms; TE =1.94 ms; flip angle = 8°; FOV = 256 mm; slice thickness = 1 mm (no gap); voxel size = 1 × 1 × 1 mm. These data were aligned in parallel to the anterior and posterior commissures and transformed into standardized space. Following source analysis (i.e., beamforming), each participant's 4.0 × 4.0 × 4.0 mm functional images were also transformed into standardized space using the transform that was previously applied to the structural MRI volume and spatially resampled.

### 2.5 MEG time-frequency transformation and statistics

Cardiac and ocular artifacts were removed from the data using signal-space projection (SSP), which was accounted for during source reconstruction (Uusitalo and Ilmoniemi, 1997). The continuous magnetic time series was divided into epochs of 2000 ms duration, from −500 ms before the onset of the flanker stimuli to 1500 ms after the onset of the stimuli. The baseline period for further analyses was defined as the window from -450 to -50 ms before the onset of the flanker stimuli to minimize any anticipation effects. Epochs containing major artifacts (e.g., eye blinks, muscle artifacts, eye saccades, swallowing, coughing) were rejected based on a fixed-threshold method, supplemented with visual inspection. Briefly, the distribution of amplitude and gradient values per participant were computed using all trials, and the highest amplitude/gradient trials relative to the total distribution were excluded. Notably, individual thresholds were set for each participant for both signal amplitude (*M* = 1178.13 fT, *SD* = 219.21) and gradient (*M* = 242.19 fT/s, *SD* = 78.40) due to differences among individuals in head size and sensor proximity, which strongly affect MEG signal amplitude. Following artifact rejection, an average of 168.69 (*SD* = 12.52) total trials per participant remained for further analysis (Congruent: *M* = 84.28 trials, *SD* = 3.59; Incongruent: *M* = 84.41 trials, *SD* = 6.79). We next tested whether the number of accepted trials was associated with age and found that age was not significantly correlated with the total number of segments retained overall, or by condition (*r_total_* = −0.11, *p* = .59; *r_congruent_* = −0.13, *p* = .48;. *r_incongruent_* = −0.061, *p* = .74).

Artifact-free epochs were transformed into the time-frequency domain using complex demodulation (resolution: 1.0 Hz, 50 ms), and the resulting spectral power estimations per sensor were averaged over trials to generate time-frequency plots of mean spectral density. These sensor-level data were normalized using the respective bin's baseline power, which was calculated as the mean power during the -450 to -50 ms baseline time period. The time-frequency windows used for imaging were determined by statistical analysis of the sensor-level spectrograms across all correct trials (congruent and incongruent) and gradiometers during the first 600 ms following stimulus onset from 1 to 50 Hz. These time and frequency windows were selected to maximize focus on the selective attention components, while minimizing the impact of other brain responses (e.g., motor) associated with each trial. To reduce the risk of false-positive results while maintaining reasonable sensitivity, a two-stage procedure was followed to control for Type 1 error. In the first stage, two-tailed paired-sample *t*-tests versus baseline were conducted on each data point and the output spectrograms of *t*-values were thresholded at *p* < .05 to define time-frequency bins containing potentially significant oscillatory deviations across all participants. In stage two, the time-frequency bins that survived the threshold were clustered with temporally and/or spectrally neighboring bins that were also below the *p* < .05 threshold, and a cluster value was derived by summing all the *t*-values of all data points in the cluster. Nonparametric permutation testing was then used to derive a distribution of cluster values and the significance level of the observed clusters (from stage one) was tested directly using this distribution (Ernst, 2004; Maris and Oostenveld, 2007). For each comparison, 10,000 permutations were computed to build a distribution of cluster values. Based on these analyses, the time-frequency windows that contained significant oscillatory events across all participants were subjected to the beamforming analysis (see “Sensor-Level Results” in the Results section).

### 2.6 MEG source imaging and statistics

Cortical activity was imaged through an extension of the linearly constrained minimum variance vector beamformer (Gross et al., 2001; Hillebrand et al., 2005; Veen et al., 1997), which employs spatial filters in the frequency domain to calculate source power for the entire brain volume. The single images were derived from the cross-spectral densities of all combinations of MEG gradiometers averaged over the time-frequency range of interest, and the solution of the forward problem for each location on a grid specified by input voxel space. This use of the cross-spectral densities is often referred to as the dynamic imaging of coherent sources (DICS) beamformer (Gross et al., 2001). Following convention, we computed noise-normalized, source power per voxel in each participant using active (i.e., task) and passive (i.e., baseline) periods of equal duration and bandwidth (Hillebrand et al., 2005). Such images are typically referred to as pseudo-*t* maps, with units (i.e., pseudo-*t*) that reflect noise-normalized power differences (i.e., active vs. passive) per voxel. MEG preprocessing and imaging were completed using BESA version 6.1. Images were derived for all correct trials combined, and separately for congruent and incongruent trial conditions.

Normalized differential source power was computed for the statistically-selected time-frequency bands (see below) over the entire brain volume per participant at 4.0 × 4.0 × 4.0 mm resolution. The resulting 3D maps of brain activity were averaged across participants to assess the neuroanatomical basis of significant oscillatory responses identified through the sensor-level analysis across all correct trials, and within each condition (i.e., congruent and incongruent). Given the focus of the study, we subtracted the congruent from the incongruent maps within each participant, per oscillatory response (e.g., theta), to derive a map of neural interference activity (i.e., a neural flanker effect). Whole-brain correlations were computed between the participant-level interference maps and chronological age to examine developmental changes in the neural responses across the whole sample, and then separately for males and females. Sex effects in the correlational maps were tested using Fisher’s *r* to *Z* transformations. All maps were smoothed with a 4 mm smoothing kernel, thresholded at a significance level of *p* < .005, and corrected for multiple comparisons using a cluster criterion requiring a minimum of at least 300 contiguous voxels, which was a conservative estimate based on the spatial smoothness of the image.

Finally, we conducted a set of exploratory analyses to examine whether the significant neural oscillatory effects identified in the primary flanker interference analyses were related to behavioral performance during the task. Linear regressions (simple path models) and, when applicable, mediation analyses were performed to identify any relationships between significant flanker-related oscillatory effects and the flanker reaction time effect. In cases of sex differences, multigroup analyses were used to simultaneously estimate effects of neural activity on behavior among males and females. Details of each analysis are provided in the Results section. Because of the exploratory nature of these analyses, we utilized bias-corrected confidence intervals based on 1,000 bootstrapped samples to more robustly detect any potential relationships between brain activity and behavior (Austin and Tu, 2004; Efron and Tibshirani, 1986; Fritz and MacKinnon, 2007). Analyses were conducted in Mplus version 8.1.

## 3 Results

### 3.1 Descriptive statistics and task behavior

Five participants were excluded during initial preprocessing due to poor performance on the task and/or technical problems during recording. An additional 13 participants were excluded due to head movement or excessively noisy MEG data. Thus, the final sample consisted of 53 participants (*M* = 13.29 years, *SD* = 1.93; 32 males; 4 left-handed). To ensure that the reduced sample did not introduce age bias in the MEG data, we once again tested whether the number of trials retained for analysis correlated with age within the evaluable sample. This showed that age was not significantly related to the number of trials included in analyses overall or by condition (*r_total_* = .14, *p* = .24; *r_congruent_* = .11, *p* = .42; *r_incongruent_* = .16, *p* = .24).

Participants in the final sample performed well on the task, achieving a mean accuracy of 97.58 % correct (*SD* = 3.58) on congruent trials, and 96.53 % correct (*SD* = 4.58) on incongruent trials. A paired-samples *t*-test revealed that average reaction times for congruent trials (*M* =677.22 ms, *SD* = 135.45) and incongruent trials (*M* =712.11 ms, *SD* = 3.58) significantly differed, *t*(52) = 7.06, *p* < .001. Thus, we observed the classic flanker effect whereby participants were slower to respond to incongruent relative to congruent trials (*M* =34.88 ms, *SD* = 35.96).

**Fig. 2:**
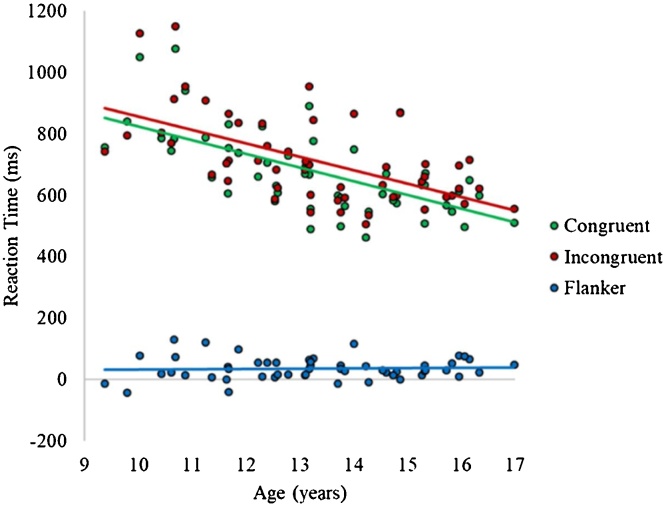
Correlations between age and reaction times to congruent and incongruent trials, and the reaction time flanker effect for the full sample.

Reaction times within each condition significantly correlated with age, such that older participants tended to respond more quickly than younger participants (see Fig. 2; *r*_congruent_ = −0.64, *p* < .001; *r*_incongruent_ = −0.59, *p* < .001). In contrast, the flanker effect did not significantly relate to age, *r* = .051, *p* = .72. Further, examination of sex effects revealed no significant differences between males and females in accuracy or reaction time within either condition, or in the flanker effect (*t*’s = -1.12 to 0.44; *p*’s = .27–.89).

### 3.2 Sensor level results

**Fig. 3:**
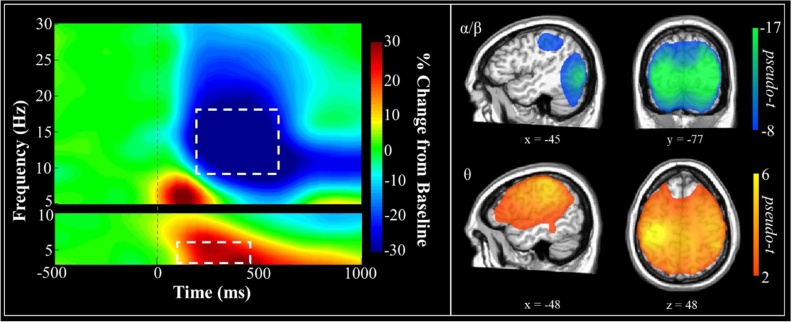
Spectrograms and source reconstructions for the combined trials (incongruent and congruent). Time-frequency decomposition and permutation-corrected statistical analyses indicated two time-frequency bins with significant responses (*p* < .05, corrected) relative to baseline during the first 600 ms period of interest. These included theta activity (3-6 Hz; bottom left) from 100-450 ms, and alpha/beta activity (9-18 Hz) from 200-600 ms. The statistical analyses included all gradiometers, but shown here are the sensors most clearly showing the response (i.e., M1122 for theta, and M2322 for alpha/beta). To the right are the grand-averaged source reconstructions per time-frequency bin across participants. As shown, theta increases were detected across a widespread network that included dorsal prefrontal and motor cortices, whereas alpha/beta and gamma responses were largely constricted to parietal and occipital cortices.

Sensor-level spectrograms for all correct trials (collapsed across congruent and incongruent conditions) were statistically examined using nonparametric permutation testing to derive the precise time-frequency bins for follow up beamforming analyses. The analyses indicated a significant increase (synchronization) of activity within the theta range (3−6 Hz) from 100−450 ms post stimulus onset. Upon visual inspection, this response was strongest in frontal and central sensors. Additionally, there was a significant decrease (desynchronization) of activity in the alpha/beta range (9−18 Hz) from 200−600 ms post-stimulus, largely distributed over central and posterior sensors. Both of the identified clusters were significant at *p* < .05 corrected (see Fig. 3). Neural activity generating these time-frequency responses was imaged for all trials combined and separately within each condition (congruent and incongruent) for each participant. Condition maps were then subtracted within each participant (incongruent – congruent) in order to examine the flanker interference effect on neural activity elicited during the task. Resultant maps were examined statistically for developmental and sex effects.

### 3.3 Functional mapping results

#### 3.3.1 Combined trials

Beamformer images per time-frequency bin were averaged across all participants and conditions. These grand-average maps revealed an early increase in theta that extended broadly across frontal, motor, and parietal cortices, with additional smaller peaks in temporal and occipital areas (Fig. 3). There were also strong decreases in alpha/beta across bilateral occipital and parietal regions, with an additional peak in the left inferior parietal area.

#### 3.3.2 Flanker interference effect

**Fig. 4:**
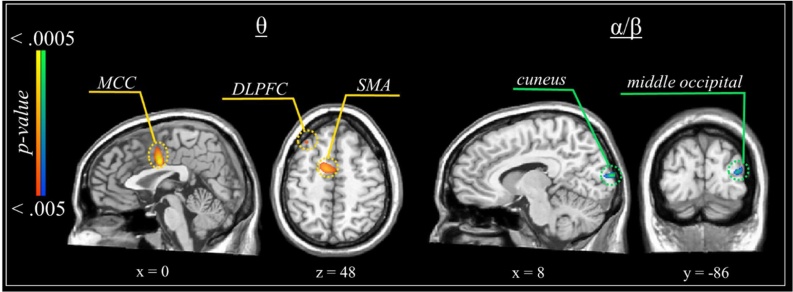
Results of a one-sample *t*-test comparing the subtracted beamformer maps (incongruent – congruent) to zero to detect significant effects of congruency on neural processing for the full sample. All maps are thresholded at *p* < .005, corrected. DLPFC = dorsolateral prefrontal cortex; MCC = middle cingulate cortex; SMA = supplementary motor area.

Beamformer images per condition and time-frequency bin were subtracted within each participant (incongruent – congruent), thereby yielding functional maps of neural flanker interference effects by participant. There were significant increases in cortical theta activity during incongruent relative to congruent trials (*p* < .005; Fig. 4). Specifically, there were significantly greater theta increases within the middle cingulate cortex (MCC) and in bilateral dorsolateral prefrontal cortex during incongruent relative to congruent trials. Additionally, the data indicated greater alpha/beta decreases (i.e., desynchronization) in the right cuneus and middle occipital gyrus during incongruent relative to congruent trials.

#### 3.3.3 Correlations with age

**Fig. 5:**
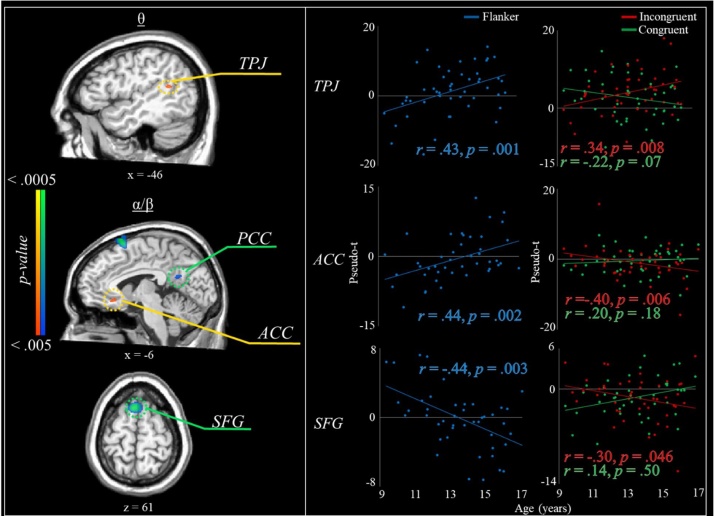
Correlations between chronological age and flanker interference maps per oscillatory response. (*Left panel*) Correlation maps showing selected significant relationships between age and theta (top) or alpha/beta (middle and bottom) interference activity. (*Right panel*) Scatterplots showing the correlations between the amplitude of the flanker interference effect at the peak voxel and age (left), or condition-specific oscillatory response amplitude at the peak voxel and age (right). Note: ACC = anterior cingulate cortex; PCC = posterior cingulate cortex; SFG = superior frontal gyrus; TPJ = temporoparietal junction.

Next, these flanker interference maps for theta and alpha/beta activity were correlated with chronological age to determine whether flanker interference effects were sensitive to maturation within the full sample. Flanker-related theta increases were associated with age in the right temporoparietal junction (TPJ), such that older youth exhibited a stronger theta interference effect (all *p*’s < .005; Fig. 5). Further examination of the condition specific effects confirmed that theta increases during incongruent trials increased with age, whereas activity during congruent trials marginally decreased with age. Interference activity in the alpha/beta range was also correlated with age in some brain areas (Fig. 5). For example, within the rostral anterior cingulate cortex (rACC) there was a positive correlation with age, indicating a decreased flanker interference effect over time. Analysis of the condition effects indicated that alpha/beta decreases during incongruent trials remained relatively constant with age, while alpha/beta decreases during congruent trials became stronger (i.e., more negative) with increasing age. In contrast, there was a negative correlation between age and alpha/beta interference activity in the left superior frontal gyrus (SFG), indicating a larger flanker effect in this region with increasing age. Condition-specific correlation maps suggested that youth exhibited stronger alpha/beta responses (i.e., more negative) with increasing age during incongruent trials, and marginally weaker alpha/beta responses with increasing age during congruent trials in the SFG. The same was true of additional peaks in the left inferior frontal gyrus, and in the posterior cingulate gyrus.

#### 3.3.4 Sex differences

**Fig. 6:**
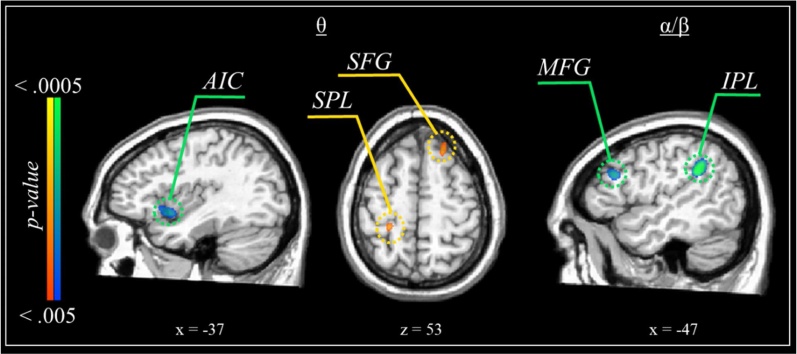
Sex differences in flanker-related interference activity. Maps are thresholded at *p* < .005, corrected. In the theta images (left and middle), warmer colors indicate clusters in which males exhibited significantly stronger theta responses relative to females, while cooler colors reflect the opposite. In the alpha/beta image (right), cooler colors indicate clusters in which males exhibited significantly stronger alpha/beta responses (i.e., decreases from baseline) relative to females. Note: AIC = anterior insula cortex; IPL = inferior parietal lobule; MFG = middle frontal gyrus; SFG = superior frontal gyrus; SPL = superior parietal lobule.

To identify neural flanker effect differences between males and females, independent samples *t*-tests were conducted on the whole-brain interference maps for theta and alpha/beta flanker activity (Fig. 6). Females exhibited a stronger theta flanker interference effect compared to males within the left anterior insula. In contrast, males had stronger flanker effects than females in the theta range within the right SFG and left superior parietal lobule. Regarding alpha/beta, males showed greater flanker interference effects in the left inferior parietal lobule, bilateral precuneus, and middle frontal gyri compared to females.

#### 3.3.5 Age by sex interactions

**Fig. 7:**
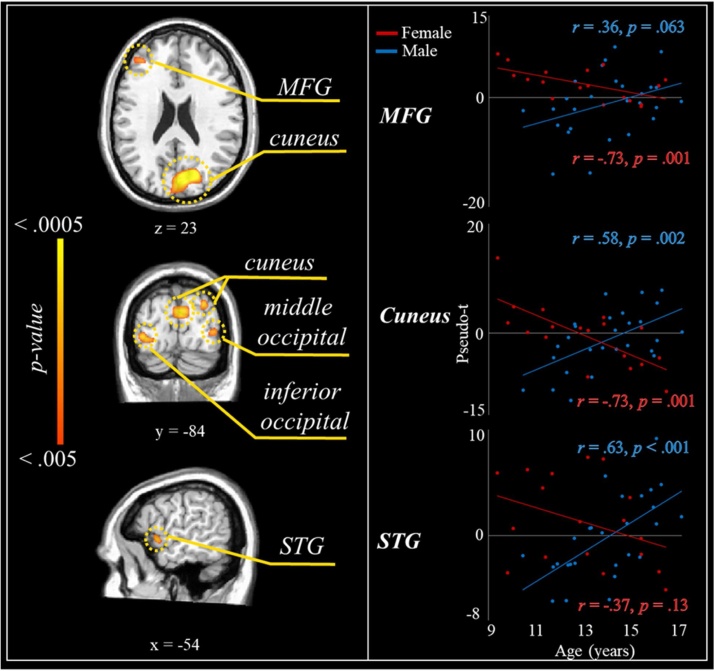
Age-by-sex interactions in flanker interference activity. (*Left panel*) Fisher’s *r* to Z maps indicating significant differences between males and females in the correlation between chronological age and alpha/beta flanker interference activity. Note that no age-by-sex interactions were observed for theta interference activity. (*Right panel*) Scatterplots showing the correlations between chronological age and flanker-related alpha/beta activity. Note: MFG = middle frontal gyrus; STG = superior temporal gyrus.

To probe age-by-sex interactions, whole-brain maps of the theta and alpha/beta flanker interference effects were correlated with age separately for males and females. The resultant maps were compared using Fisher’s *r* to Z transformations. There were no significant age-by-sex interactions at the *p* < .005 level for flanker-related theta activity. However, in the alpha/beta range, there were significant age-by-sex interactions in flanker interference activity in the left middle frontal (*Z* = 3.30), superior temporal (*Z* = 3.41), and left lingual gyrus (*Z* = 3.77), and in the right cuneus (two clusters; *Z*’s = 4.84 and 3.56), precuneus (*Z* = 3.30), and middle occipital gyrus (*Z* = 3.31; Fig. 7).

When viewing the age correlations within condition-specific maps, males generally showed a trend toward weaker desynchronizations (i.e., weaker responses) as a function of age during incongruent trials, and greater desynchronization (i.e., stronger responses) during congruent trials. Conversely, females tended to exhibit greater alpha/beta desynchronization (i.e., stronger responses) during incongruent trials, with little-to-no change in alpha/beta activity during congruent trials across development. A complete summary of all significant effects can be found in Table 1.

**Table 1:** Coordinates of the peak response in each significant neural flanker effect cluster.

Region/Effect of Interest	Frequency	X	Y	Z	Statistic
***Flanker Effect (Incongruent > Congruent)***					***t***
MCC	θ	−10	−6	34	4.35
Right DLPFC	θ	16	16	63	3.31
Left DLPFC	θ	−29	20	39	3.29
Left SMA	θ	−11	−3	47	3.50
Left middle temporal gyrus	θ	−38	−56	21	3.26
Right cuneus	α/β	6	−96	13	−3.91
Right middle occipital gyrus	α/β	33	−89	10	−3.85

***Flanker Effect: Correlations with Age***					***r***
Left TPJ	θ	−40	−48	15	.43
ACC	α/β	−13	27	−7	.43
Left SFG	α/β	−5	13	59	−.45
PCC	α/β	−11	−54	24	−.45

***Flanker Effect: Differences by Sex (Male > Female)***					***t***
Left anterior insula cortex	θ	−39	9	−7	−3.35
Right MFG	θ	20	29	51	3.41
Left IPL	θ	−25	−36	56	3.93
Left IPL	α/β	−55	−43	29	−4.02
Right precuneus	α/β	14	−44	52	−4.10
Left precuneus	α/β	−9	−57	36	−3.45
Right MFG	α/β	22	32	39	−3.39
Left MFG	α/β	−43	22	25	−3.73

***Flanker Effect: Age x Sex Interactions***					***Z***
Right precuneus	α/β	2	−79	48	3.39
Right cuneus	α/β	12	−69	17	4.86
Right cuneus	α/β	16	−96	5	3.66
Left lingual	α/β	−30	−94	−7	3.88
Right middle occipital gyrus	α/β	37	−86	4	3.32
Left MFG	α/β	−36	43	25	3.31
Left STG	α/β	−50	5	−3	3.20

*Note*: All test statistics are significant at the *p* < .005 level; All coordinates are in Talairach space. Note: ACC = anterior cingulate cortex; DLPFC = dorsolateral prefrontal cortex; IPL = inferior parietal lobule; MCC = middle cingulate cortex; MFG = middle frontal gyrus; PCC = posterior cingulate cortex; SFG = superior frontal gyrus; SMA = supplemental motor area; STG = superior temporal gyrus; TPJ = temporoparietal junction.

#### 3.3.6 Relationships to behavior

Finally, we performed exploratory analyses to examine the relationship between interference related neural oscillatory responses and the reaction time (RT) flanker effect. First, we examined the brain regions that showed a significant flanker effect across the whole sample (i.e., right DLPFC, MCC, cuneus and occipital regions). We found that increased flanker-related alpha/beta activity in the right cuneus predicted decreases in the RT flanker effect (β = −.48; b = −2.79, 95 % CI [−4.69, −.48]), whereas increased alpha/beta activity in middle occipital gyrus was associated with an increased RT flanker effect (β = .49; b = 5.02, 95 % CI [.50, 8.78]). Next, we probed brain regions exhibiting developmental effects and sex differences. We only detected one significant association, namely that flanker-related alpha/beta interference activity within the superior frontal gyrus (identified in whole-brain correlations with age; Fig. 7) was associated with the RT flanker effect (β = −.72; b = −6.59, 95 % CI [−11.73, −2.41]).

**Fig. 8:**
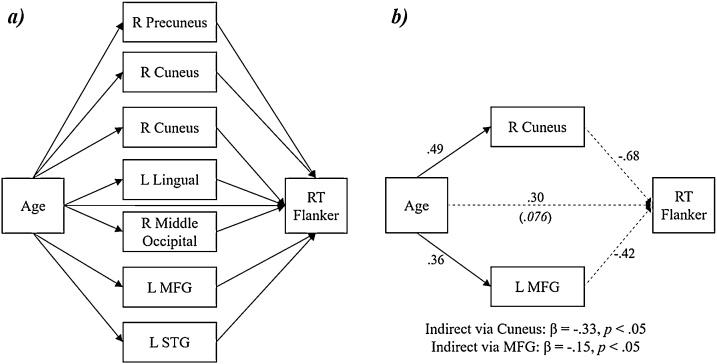
Mediation analysis interrogating the relationship between behavior and brain regions exhibiting age-by-sex interactions, separately for females and males. *a*) Depiction of the model tested using a multi-group approach; all neural variables were allowed to freely correlate, though the relationships are not shown in the figure for simplicity. *b*) Summary of significant mediations detected in the analysis among males. All reported coefficients are standardized. The italicized value in parentheses reflects the total effect of age on the reaction time (RT) flanker effect, which was not statistically significant. Significant indirect effects identified by bias-corrected bootstrapped confidence intervals are listed below the model. Solid lines signify statistically significant relationships at the *p* < .05 level; dotted lines designate non-significant relationships. Note: R = right; L = left; MFG = middle frontal gyrus; STG = superior temporal gyrus; RT flanker = flanker effect on reaction time (incongruent – congruent).

We did not detect any significant mediating effects among females. However, there were two brain regions that mediated the effect of age on the RT flanker effect among males (Fig. 8b). Age was significantly associated with alpha/beta flanker-related activity in one peak located in the right cuneus (β = .49; b = 2.06, 95 % CI [.23, 3.42]) and another in the left middle frontal gyrus (β = .36; b = 1.23, 95 % CI [.35, 2.42]). Further, activity in the right cuneus (β = −.68; b = −3.25, 95 % CI [−5.80, .10]) and in the left middle frontal gyrus (β = −.42; b = −2.50, 95 % CI [−6.02, .32]) trended toward predicting the flanker RT effect, with moderate-to-large effect sizes. Altogether, the data show that as a function of age, males tend to exhibit weaker alpha/beta responses during incongruent trials, and stronger responses during congruent trials within the right cuneus and left middle frontal gyrus. This shift in neural interference effects across development subsequently predicts reductions in behavioral interference effects.

## 4 Discussion

The present study investigated the neural oscillatory dynamics underlying selective attention abilities in typically developing youth. Our key findings were that the neural flanker interference effect was reflected by stronger theta and alpha/beta responses within critical areas for visual selective attention and cognitive control. Moreover, we found developmental and sex-specific effects on flanker-related oscillatory activity, despite similar task performance between males and females. Behaviorally, youth performed quite well overall, and participants showed the expected, classic flanker effect; youth were quicker to respond to congruent relative to

incongruent trials (e.g., Embury et al., 2019; McDermott et al., 2017; Wiesman et al., 2020). Reaction times were developmentally sensitive, with older youth typically responding faster than their younger peers on both congruent and incongruent trial types.

When we examined the neural oscillatory dynamics serving task performance across the sample, we saw distributed increases in frontoparietal theta activity along with robust decreases in occipital and parietal alpha/beta activity, all of which corroborates previous research in adult populations (Driver and Frackowiak, 2001; Embury et al., 2018; Jiang et al., 2017; Lew et al., 2020, 2018; Mazaheri et al., 2014; McDermott et al., 2017; Saenz et al., 2002). Such regions generally exhibited neural flanker effects, including greater theta increases within the MCC and bilateral DLPFC, along with stronger alpha/beta decreases in the right cuneus and middle occipital gyrus during incongruent relative to congruent trials. In other words, all of these regions, which critically contribute to the top-down control of selective attention processes (Cavanagh and Frank, 2014; Petersen and Posner, 2012; Rihs et al., 2007; Spooner et al., 2019), exhibited a response pattern consistent with the classic flanker effect. Importantly, flanker-related neural activity within the DLPFC was related to the RT flanker effect, such that greater neural interference was associated with greater behavioral interference. The DLPFC is frequently implicated in high-order cognition and performance, especially during attention tasks (Amso and Scerif, 2015; Couperus, 2011; Pozuelos et al., 2014).

In addition to the group-level interference effects, we saw age-related changes in flanker-related oscillations within several critical regions implicated in cognitive control and attention. For example, increasing age was associated with a larger flanker effect (i.e., stronger oscillatory responses during incongruent relative to congruent trials) within the right TPJ (theta) and the left SFG (alpha/beta). Conversely, increasing age was associated with a reduced flanker effect (i.e., weaker oscillatory responses to incongruent relative to congruent trials) in the alpha/beta range within the rACC. Each of these regions has been commonly associated with distractor effects in prior literature; for instance, the TPJ is part of the ventral attention system and contributes to bottom-up control of attention (Doesburg et al., 2016; Lew et al., 2018; Parks and Madden, 2013). Conversely, portions of superior frontal cortex are implicated in top-down attentional control systems that tend to come online and refine with increasing age (McDermott et al., 2017; Parks and Madden, 2013; Petersen and Posner, 2012; Segalowitz and Davies, 2004). Finally, activity within the rACC is frequently studied in response to congruency effects and is believed to be a critical hub in conflict signaling and performance monitoring (Botvinick et al., 2004; Danckert et al., 2000; McDermott et al., 2017). Activation within each of these three regions assessed using functional MRI is known to mature and refine over time, ultimately contributing to improvements in high-order cognitive abilities (Bunge et al., 2002; Casey et al., 2005; Dumontheil, 2016; Huyser et al., 2011; Rueda et al., 2004; Vijayakumar et al., 2014). Thus, the present study supports these previous findings and adds critical new data on the developmental trajectory of the neural oscillatory dynamics underlying selective attention processing in these regions.

Interestingly, we also detected multiple sex-specific effects in flanker-related neural oscillatory activity in both theta and alpha/beta bands. On average, females exhibited greater interference within the left anterior insula (theta), whereas males exhibited greater interference activity within multiple frontoparietal regions implicated in visual attentional control (theta and alpha/beta). The anterior insula is commonly implicated in conflict processing and high-level cognitive control (McDermott et al., 2019; Sauseng et al., 2007). Increased cortical activity in the anterior insula among females, coupled with the increased frontoparietal activity among males, may be indicative of different neurocognitive strategies (e.g., Doyon and Benali, 2005; Li et al., 2012; Taylor et al., 2019), or possibly different biological mechanisms serving attention between males and females, with each group relying on slightly different configurations of attentional networks based on differing developmental trajectories (Petersen and Posner, 2012; Pozuelos et al., 2014; Rueda et al., 2004). Notably, these findings add to a growing body of research showing sexually-divergent patterns of neurophysiological responses serving diverse attentional processes, including visuospatial attention (Fung et al., 2020; Killanin et al., 2020), sustained attention (Taylor et al., 2019), and more broadly, resting state measures of functional networks serving attention (de Lacy et al., 2019).

Moreover, we detected multiple age-by-sex interactions in the neural flanker effect. Males exhibited positive associations between neural interference effects and age, while females showed the opposite pattern. Importantly, flanker-related alpha/beta activity in the cuneus and middle frontal gyrus mediated the effect of age on the behavioral flanker effect among males, but not females, with decreases in neural interference predicting improved behavioral performance. The cuneus is commonly featured in attention networks and has been linked to directed attention processes, particularly when activation is coupled with structures like the anterior insula and frontoparietal regions (Doesburg et al., 2016; Hahn et al., 2006; Simpson et al., 2011; Vance et al., 2007). Moreover, posterior alpha oscillatory activity is thought to support inhibition of irrelevant or distracting visual information (Händel et al., 2010; Heinrichs-Graham and Wilson, 2015; Payne et al., 2013; Proskovec et al., 2019; Wilson et al., 2017). The present findings might suggest that adolescent males shift to different strategies of selective attention control as a function of age, thereby supporting better behavioral performance in the face of distracting information via newly matured top-down mechanisms over time. Overall, the novel sex-specific effects reported in the present study suggest differential maturation of neural oscillatory dynamics serving selective attention abilities between typically developing males and females.

Prior work has shown sexual divergence in the neural oscillatory mechanisms underlying higher-order cognitive abilities in developing youth (Blakemore et al., 2010; Blakemore and Choudhury, 2006; Embury et al., 2019; Taylor et al., 2020). For example, Taylor et al. (2020) showed that males relative to females tended to have prolonged developmental trajectories of theta oscillatory activity serving abstract reasoning abilities across a distributed frontoparietal network. Likewise, a study of working memory abilities showed that females had larger alpha decreases within right inferior frontal areas as a function of age during initial memory encoding, whereas males had greater alpha increases with age within parietal, occipital, and cerebellar regions during later maintenance of working memory (Embury et al., 2019). Each of these studies shed light on the developmental sensitivity of neural oscillatory dynamics serving high-order cognition. However, in each study, developmental findings were confined to a single oscillatory band; the present study found distributed developmental and sex-specific effects across both the alpha/beta and theta bands, each of which is thought to serve putatively unique functions. Specifically, theta oscillatory activity is believed to support long-range neuronal communication and coordination of information processing (Colgin, 2013; Herrmann et al., 2016), whereas alpha/beta oscillatory activity, particularly in posterior regions, tends to be related to inhibition of irrelevant visual information (Händel et al., 2010; Payne et al., 2013). More anteriorly, beta is often associated with motor function (Heinrichs-Graham et al., 2020, 2018b, 2016, 2014). The current study examining the neural oscillatory dynamics serving selective attention may have been uniquely sensitive to the development of multiple integrative neurocognitive systems given the cross-cutting nature of selective attention and inhibitory control required to perform the classic flanker task.

Before closing, we must note several limitations of the current study. First, we lost a number of participants during MEG preprocessing due to excessively noisy data, commonly linked to muscle and eye artifacts. Youth relative to adults are already prone to increased movement during neuroimaging scans; coupled with a cognitively demanding task, participants frequently clench their jaws, furrow their brows, or move around in the scanner during task performance (Gavin et al., 2008). It is possible that with the development of more advanced artifact removal procedures we would have been able to include more participants in our final analyses, as overall task performance was excellent for the majority of those excluded in the present study. Second, the flanker task was adapted for use in MEG and exploring neural oscillatory dynamics, meaning that trials were generally longer than is typically seen in some event-related potential studies (e.g., Gavin et al., 2019; Meyer et al., 2014). Longer trials typically contribute to higher overall accuracy rates, as was seen in the present study. Because of the low number of incorrect trials, we were unable to examine neural oscillatory dynamics during error trials. Finally, the present study examined development in a cross-sectional rather than a longitudinal design. Thus, we cannot make any claims that developmental trends in the oscillatory dynamics directly cause changes in behavior. Future work should consider examining the evolution of neural oscillatory dynamics serving selective attention over time within individuals, and try to determine whether changes in those dynamics causally predict changes in behavioral performance.

## 5 Conclusions

To conclude, our findings indicated distributed sex-specific and developmental effects of flanker interference-related neural oscillations, which may suggest a complex, interwoven pattern of maturation across multiple neurocognitive brain networks underlying selective attention. Both males and females exhibited refinement of activity within a number of brain regions that are known to support higher-order cognition, including the anterior insula, TPJ, frontoparietal regions, and the rACC. Importantly, males showed age-related changes in neural interference within the cuneus and middle frontal gyrus, which then predicted reductions in behavioral interference. These data suggest that males tended to fine-tune processes serving the inhibition of distracting stimuli in a different manner than their female peers, ultimately supporting improved behavioral performance in the face of interference.

## Funding

This work was supported by the 10.13039/100000001National Science Foundation (#1539067); and the 10.13039/100000002National Institutes of Health (R01-MH121101, R01-MH116782, R01-MH118013, P20-GM103472, R01-EB020407). The funders had no role in the study design, collection, analysis, or interpretation of data, nor did they influence writing the report or the decision to submit this work for publication.

## Data availability

All data are available upon request to the corresponding author (TWW). Data will be made publicly available upon study completion.

## Declaration of Competing Interest

All authors have no conflicts of interest to disclose.
